# DNA stable isotope probing on soil treated by plant biostimulation and flooding revealed the bacterial communities involved in PCB degradation

**DOI:** 10.1038/s41598-022-23728-2

**Published:** 2022-11-10

**Authors:** Lorenzo Vergani, Francesca Mapelli, Magdalena Folkmanova, Jakub Papik, Jan Jansa, Ondrej Uhlik, Sara Borin

**Affiliations:** 1 Department of Food, Environmental and Nutritional Sciences, University of Milan, Milan, Italy; 2 Departement of Biochemistry and Microbiology, Faculty of Food and Biochemical Technology, University of Chemistry and Technology Prague, Prague, Czech Republic; 3 Laboratory of Fungal Biology, Institute of Microbiology, Czech Academy of Sciences, Prague, Czech Republic

**Keywords:** Soil microbiology, Microbial ecology, Environmental biotechnology

## Abstract

Polychlorinated biphenyl (PCB)-contaminated soils represent a major treat for ecosystems health. Plant biostimulation of autochthonous microbial PCB degraders is a way to restore polluted sites where traditional remediation techniques are not sustainable, though its success requires the understanding of site-specific plant–microbe interactions. In an historical PCB contaminated soil, we applied DNA stable isotope probing (SIP) using ^13^C-labeled 4-chlorobiphenyl (4-CB) and 16S rRNA MiSeq amplicon sequencing to determine how the structure of total and PCB-degrading bacterial populations were affected by different treatments: biostimulation with *Phalaris arundinacea* subjected (PhalRed) or not (Phal) to a redox cycle and the non-planted controls (Bulk and BulkRed). Phal soils hosted the most diverse community and plant biostimulation induced an enrichment of Actinobacteria. Mineralization of 4-CB in SIP microcosms varied between 10% in Bulk and 39% in PhalRed soil. The most abundant taxa deriving carbon from PCB were Betaproteobacteria and Actinobacteria. Comamonadaceae was the family most represented in Phal soils, Rhodocyclaceae and Nocardiaceae in non-planted soils. Planted soils subjected to redox cycle enriched PCB degraders affiliated to Pseudonocardiaceae, Micromonosporaceae and Nocardioidaceae. Overall, we demonstrated different responses of soil bacterial taxa to specific rhizoremediation treatments and we provided new insights into the populations active in PCB biodegradation.

## Introduction

Polychlorinated biphenyl (PCB)-contaminated soils represent a major environmental source of persistent organic pollutants (POPs) worldwide, with important concerns for ecosystems and public health^1,2^. Microbial communities providing PCB biodegradation abilities through reductive dichlorination and aerobic pathways are the main agents of natural attenuation in historically contaminated environments, hence, when properly biostimulated, constitute a natural resource for the reclamation of polluted soils^3^. Several studies have reported that the “rhizosphere effect” exerted by plant root exudation and root deposition on the soil microbiome has a positive influence on the degradation of POPs, including PCBs, as a consequence of the induction of microbial degradation pathways by secondary plant metabolites^4–7^. Therefore, plant-driven biostimulation of PCB-degrading soil microbial populations (i.e., rhizoremediation) has been identified as a way to accelerate the natural attenuation process in the attempt to restore ecosystem services in those sites where the implementation of traditional remediation techniques based on soil removal and incineration are not sustainable due to costs and environmental impact, such as extended agricultural areas^8^. However, root growth and exudation profile, soil microbial community, and their interdependent relationships may undergo temporal and spatial variations depending on the plant species and phenological growth stage along with soil edaphic conditions and contamination profile^9–11^.

In a previous, 18-month rhizoremediation study^12^ we reported how ten plant species combined with soil treatments differentially affected the structure and activity of the soil microbial community and ultimately the degradation rate of different PCB congeners in a weathered contaminated soil. In the present work we focused on the soils that during this rhizoremediation trial had been i) biostimulated with reed canary grass (*Phalaris arundinacea* L.) and/or ii) subjected to periodic flooding with the aim to induce a redox cycle that would allow both reductive dichlorination and aerobic degradation of PCBs by autochthonous microorganisms^13^. These treatments were chosen because they promoted the reduction of the concentration of tri-, tetra- and penta-chlorinated PCB in the original soil after 18 months^12^. We hypothesized that the combined or independent application of plant biostimulation and cyclic oxic-anoxic conditions given by the periodical flooding would shape the soil bacterial communities and would enrich different populations among the autochthonous PCB degraders present in this historically polluted soil. Therefore, we applied DNA-based stable isotope probing (SIP) incubation combined with 16S rRNA amplicon sequencing to determine how the plant growth and the redox cycles affected the structure of bacterial communities and their diversity and, in particular, the metabolically active bacteria potentially involved in PCB degradation. SIP has been successfully employed in bioremediation-oriented studies to detect bacterial taxa able to incorporate carbon from diverse organic pollutants^14,15^. Compared to other molecular techniques, this approach allows to directly link metabolic capability to phylogenetic information within a microbial community by tracking isotopically labeled carbon into the DNA, and therefore in phylogenetic biomarkers such as the 16S rRNA gene, only in actively duplicating cells. This technology enables the identification of only the organisms active in the utilization of a specific substrate, requiring less sequencing effort than a full metagenomic analysis of the total community^15^. To the best of our knowledge, whereas the non-chlorinated biphenyl backbone is generally considered a model molecule for PCB mineralization, only one study compared the results obtained from SIP incubation of sediment samples with ^13^C-labeled biphenyl or ^13^C-4-chlorobiphenyl (4-CB)^16^. This implies a lack of knowledge on the metabolization of chlorinated congeners in environmental matrices and especially in soils differentially biostimulated. To overcome this limitation and target more specifically the PCB biodegradation, in this work we used ^13^C-labeled 4-chlorobiphenyl as a metabolic tracer.

## Materials and methods

### Soil samples

Samples of soil were collected at the end of an 18-month rhizoremediation trial performed in greenhouse conditions. The trial was conducted on former agricultural soils from the National Priority Site for remediation (SIN) Brescia-Caffaro^17^ which were contaminated by PCBs but also PCDDs, PCDFs, DDT and its isomers, metalloids, and metals (mainly As and Hg)_._ The experimental set-up, conditions, and applied treatments are described in detail in our previous publication^12^. In this work, we used soils collected from four different treatments: biostimulation with *Phalaris arundinacea* (Phal) and non-planted control (Bulk); biostimulation with *Phalaris arundinacea* subjected to periodic soil flooding obtaining an oxic-anoxic (redox) cycle (PhalRed) and non-planted control subjected to the same redox cycle (BulkRed). Each treatment was performed on the same homogeneous original batch of soil in separate pots in three biological replicates.

### Stable isotope probing (SIP) incubation

We set up SIP microcosms for three incubation times: seven (D_07_), twenty-one (D_21_) and twenty-eight (D_28_) days. Triplicate microcosms were established for each time point and soil treatment, corresponding to each original biological replicate of the four rhizoremediation treatments, totaling 36 microcosms. The set-up procedure was as follows: 10 µl of ^13^C-labeled 4-chlorobiphenyl (4-CB, 99% ^13^C, Alsachim) in acetone solution (50 mg/ml) were spiked on 0.2 g of quartz sand placed at the bottom of 100-ml sterile serum bottles to have a final quantity of 0.5 mg of 4-CB per microcosm. After acetone evaporation, 2 g of soil were added to each microcosm, manually mixed with the sand, and supplemented with 200 µl of basal mineral salt solution^18^. The microcosms were sealed and placed at 25 °C in the dark, to avoid the proliferation of photosynthetic microorganisms, until destructive harvesting when the content was stored at -80 °C. Parallel incubation with unlabeled 4-CB was carried out for each microcosm as a control.

### Isotopic analysis of headspace CO_2_

At each harvest time point, the % of O_2_ and CO_2_ in the headspace of each microcosm was measured using a CheckPoint II—Portable Headspace Analyzer (Dansensor). For the determination of CO_2_ isotopic composition, 500 μl of the headspace gas was sampled with a gas-tight syringe from labeled microcosms and transferred into helium-filled borosilicate vacutainers. The isotopic composition of headspace CO_2_ was analyzed by Gasbench II device equipped with a cryotrap coupled to a Delta V Advantage isotope ratio mass spectrometer (ThermoFisher Scientific, Bremen, Germany). The mineralization of ^13^C-labeled 4-chlorobiphenyl was calculated stoichiometrically based on the amount of ^13^CO_2_ evolved^19^.

### DNA extraction and isopycnic separation

Basing on the results of the isotopic CO_2_ analysis, the metagenomic DNA was extracted from D_21_ and D_28_ SIP microcosms and the initial, non-incubated, time zero (T_0_) soil samples using the FastDNA™ SPIN Kit for Soil (MP Biomedicals) and purified with the Genomic DNA Clean & Concentrator™ (Zymo Research) following the manufacturer’s instructions. D_07_ samples were excluded from DNA extraction because no ^13^C-labelled CO_2_ was retrieved in the microcosms’ headspace. DNA concentration was measured with a Qubit™ fluorometer (Thermo Scientific) and all samples were adjusted to a final concentration of 25 ng/µl.

For isopycnic separation, 40 µl (1,000 ng) of DNA were placed into a Quick-Seal™ centrifuge tube (Beckman) filled with cesium trifluoroacetate (CsTFA) pre-diluted to a concentration of 1.62 g/ml. Ultracentrifugation was run for 72 h at 145,000 × *g*, 24 °C, using an Optima™ MAX-XP Ultracentrifuge with TLN 100 rotor (Beckman). After ultracentrifugation, each gradient was fractionated into fractions of 100 μL using a Beckman Fraction Recovery System (Beckman) and a Harvard Pump 11 Plus Single Syringe (Harvard Apparatus) by replacement with water at a flow rate of 400 μL/min. To ensure reproducibility and accuracy, each isopycnic centrifugation and gradient fractionation were run in parallel with two blanks (water instead of DNA), then the buoyant density (BD) of each gradient-recovered fraction was inferred by measuring the refractive index of fractionated blank samples with a Digital Hand-held Refractometer (Reichert Analytical Instruments). DNA was retrieved from CsTFA by isopropanol precipitation with glycogen^18^ and stored at -20 °C.

The distribution of bacterial DNA across the density gradients was assessed by quantitative PCR (qPCR) targeting the 16S rRNA gene using primers 357F (3’-CCCTACGGGAGGCAGCAG-5’) and 907R (3’-CCGTCAATTCCTTTGAGTTT-5’) as previously described^20^. In each gradient, fractions that were determined to contain ^13^C-labeled DNA based on 16S rRNA gene copies distribution as a function of buoyant density (BD) were combined into pools. Equivalent pools were prepared for controls incubated with unlabeled substrates and were subjected to the same downstream analyses as ^13^C-labeled DNA to identify any background contamination. Fractions corresponding to the main 16S rRNA peak containing ^12^C-DNA of each gradient from both labeled and unlabeled SIP incubations and from initial, non-incubated T_0_ soils were also pooled and analyzed.

### 16S rRNA gene sequencing and data analysis

We applied Illumina MiSeq sequencing to the pooled-DNA samples after amplification using primers 515F (5’-GTGYCAGCMGCCGCGGTAA-3’) and 926R (5’-CCGYCAATTYMTTTRAGTTT-3’) targeting the V4-V5 hypervariable region of the 16S rRNA gene^21^. Reactions were performed at the Core Facility for Nucleic Acid Analysis, University of Alaska Fairbanks (USA). The 16S rRNA gene amplicon sequence data were processed using the DADA2 pipeline^22^ in R software with a few modifications to the DADA2 SOP. In brief, the primer sequences were trimmed off or, if absent, the whole read was discarded. In order to manage the lower quality towards read ends, forward and reverse reads were truncated to the length of 248 and 176 bp, respectively. The values were calculated as the average positions where 75% of reads had a quality score >  = 25 while ensuring a hypothetical minimum of 25 bp overlap between the paired reads. Filtering was based on the reads quality using the following parameters: maxN = 0, maxEE = 2, truncQ = 2. After dereplication and application of DADA2-based removal of sequencing errors, denoised forward and reverse reads were merged and chimeric sequences were removed using the method = "pooled". Based on the analysis of the mock community, which consisted of three bacterial strains (i.e., *Rhodococcus* sp. 3B12, ENA accession LT978383; *Acinetobacter* sp. P320 ENA accession LT838128; *Bacillus* sp. P28, ENA accession LT838114) that were amplified in parallel with the DNA samples, the sequences which differed by one base were clustered together and the most abundant sequence from each of the clusters was considered as the valid one. Taxonomy was assigned by using Silva v138^23^ to create a database of amplicon sequence variants (ASVs). The sequence reads were deposited in the NCBI SRA database under the BioProject ID: PRJNA809320.

Prior to further analysis, sequencing data were rarefied at 4,000 reads per sample to ensure the comparability between samples. A principal coordinate analysis (PCoA) was used to assess the phylogenetic β-diversity based on Bray–Curtis distance matrix on the normalized (log transformed) ASV table. Significant differences in bacterial community composition were investigated by canonical analysis of principal coordinates (CAP) and permutational multivariate analysis of variance (PERMANOVA), according to the factors ‘biostimulation’ (levels: ‘planted’, ‘no plant’), ‘redox’ (levels: ‘redox’, ‘no redox’), ‘treatment’ (levels: ‘Phal’, ‘Bulk’, ‘PhalRed’, ‘BulkRed’), ‘time’ (levels: T_0_, D_21_, D_28_) and ‘treatment’ and ‘time’ interaction. PERMDISP analysis was implemented prior to CAP and PERMANOVA to test the homogeneity of the data dispersion. Statistical analyses were conducted in PRIMER v. 6.1, PERMANOVA + for PRIMER routines^24^. Alpha-diversity indices (i.e., Shannon diversity and dominance) were calculated using the PAST software^25^ and their statistical difference was evaluated using the R software version 4.0.2^26^.

To identify the bacterial taxa incorporating ^13^C from ^13^C-labeled 4-CB, we summed the number of sequences for each ASV present in each of the three ^13^C-labeled replicate samples and made a comparison with the respective unlabeled control. Only ASVs that were at least ten-fold more abundant in the ^13^C-DNA pools were considered to have incorporated ^13^C.

Sequencing results of pools of fractions corresponding to the main 16S rRNA peak of each control DNA gradient from T_0_ and D_21_ / D_28_ unlabeled SIP incubations were analyzed to characterize the community dynamics among different soil treatments and over time.

## Results

### Evolution of labeled CO_2_ and 4-chlorobiphenyl mineralization

**Figure 1:**
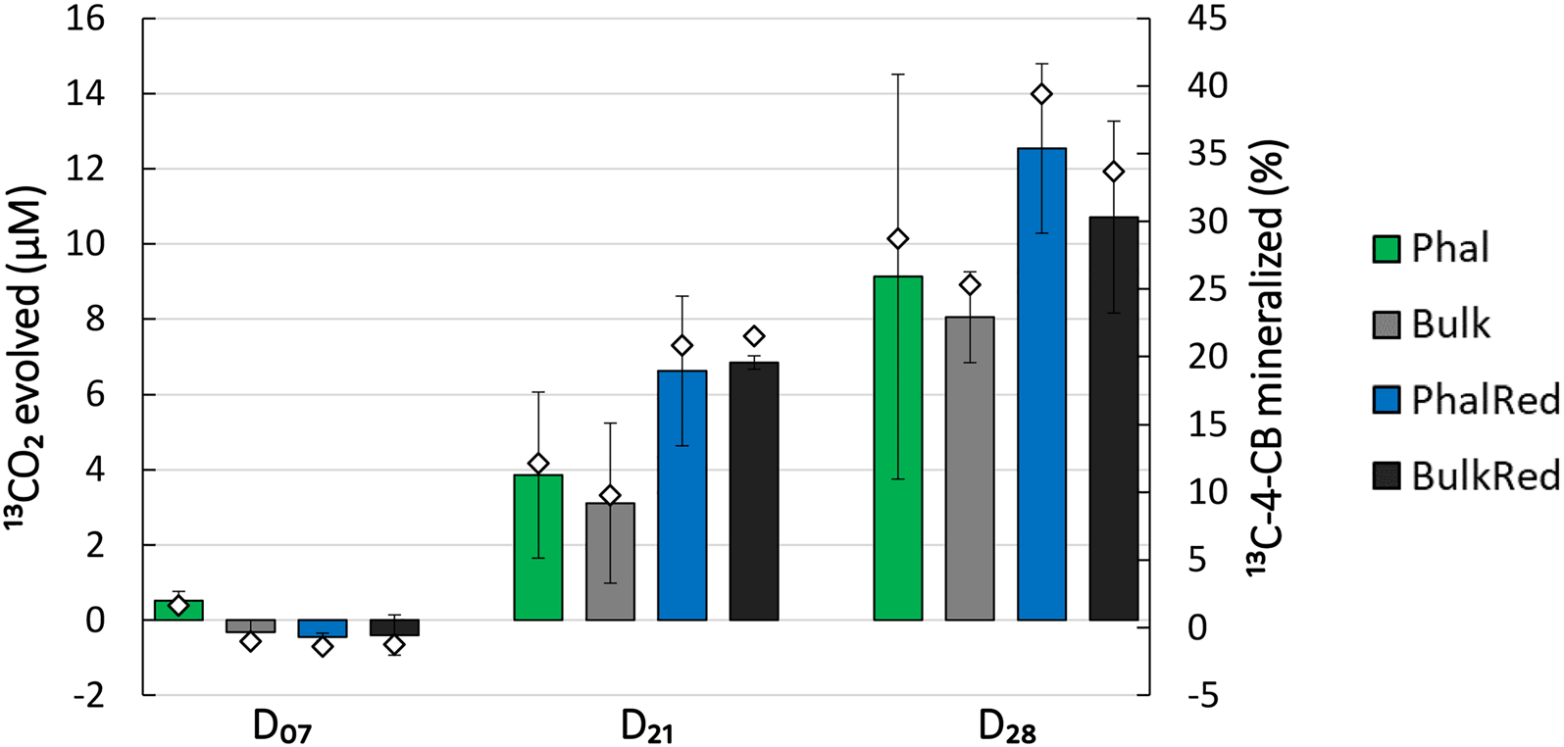
Amounts of ^13^CO_2_ (bars, left axis) evolved after 7, 21 and 28 days of soil incubation with ^13^C-4-chlorobiphenyl and percentage amount of original substrate that was converted to ^13^CO_2_ (white diamonds, right axis). Error bars represent standard deviations for ^13^CO_2_ evolved.

Results showed that after seven days (D_07_) of incubation there was no evolution of ^13^CO_2_ in the microcosms supplemented by ^13^C 4-CB, while it was detected after twenty-one days (D_21_) and increased at the end of SIP incubation (D_28_) (Fig. 1, Supplementary Table S1). For this reason, D_07_ samples were excluded from further processing and analysis. Mineralization of 4-CB was calculated from the concentration of ^13^CO_2_ and depletion of the labeled substrate occurred in all D_21_ and D_28_ microcosms and varied between 10% in Bulk soil and 22% in BulkRed soil microcosms at D_21_ and reached up to 39% for the PhalRed soil at D_28_. Even though planted and flooded soils seemed to support a higher ^13^C 4-CB mineralization rate (Fig. 1), no significant differences (*p *> 0.05, Student *t-*test) were observed among treatments, possibly because of the intrinsic variability of soil samples and the limited number of biological replicates.

Soil samples from D_21_ and D_28_ were therefore selected for labeled DNA isolation and further analysis to identify ^13^C-labeled bacterial populations.

### Isolation of labeled DNA

Following density gradient ultracentrifugation and fractionation of DNA, we performed 16S rRNA gene qPCR analyses to assess DNA distribution in the CsTFA gradients (Supplementary Table S2 A-F). Gradients from all D_28_ samples presented a small 16S rRNA peak corresponding to heavy fractions containing labeled DNA in addition to samples Bulk 3 and BulkRed 2 from D_21_. Thus, we considered only D_28_ samples for further analyses. A main 16S rRNA peak containing unlabeled DNA was observed at buoyant density of 1.60 g/mL, while ^13^C-DNA-containing fractions were identified at BDs between 1.61 and 1.64 g/mL. Minor quantities of 16S rRNA gene (Supplementary Table S2 A-F) were detected at the same BDs in samples from parallel incubations with unlabeled 4-CB, that were analyzed as controls.

### Diversity of soil bacterial communities

A total of 10,921,966 high-quality paired-end reads with an average length of 352 bp were obtained from 16S rRNA amplicon sequence data spanning all samples and replicates. Taxonomy assignment resulted in a database of 21,185 unique ASVs. After rarefaction to 4,000 reads per sample, the resulting ASV table was composed of 21,167 ASVs. Samples that were below the rarefaction threshold were excluded from the dataset (Supplementary Table S3 A-B). ASVs representing less than 0.005% of the total bacterial community were cut off prior to further analyses.

**Figure 2:**
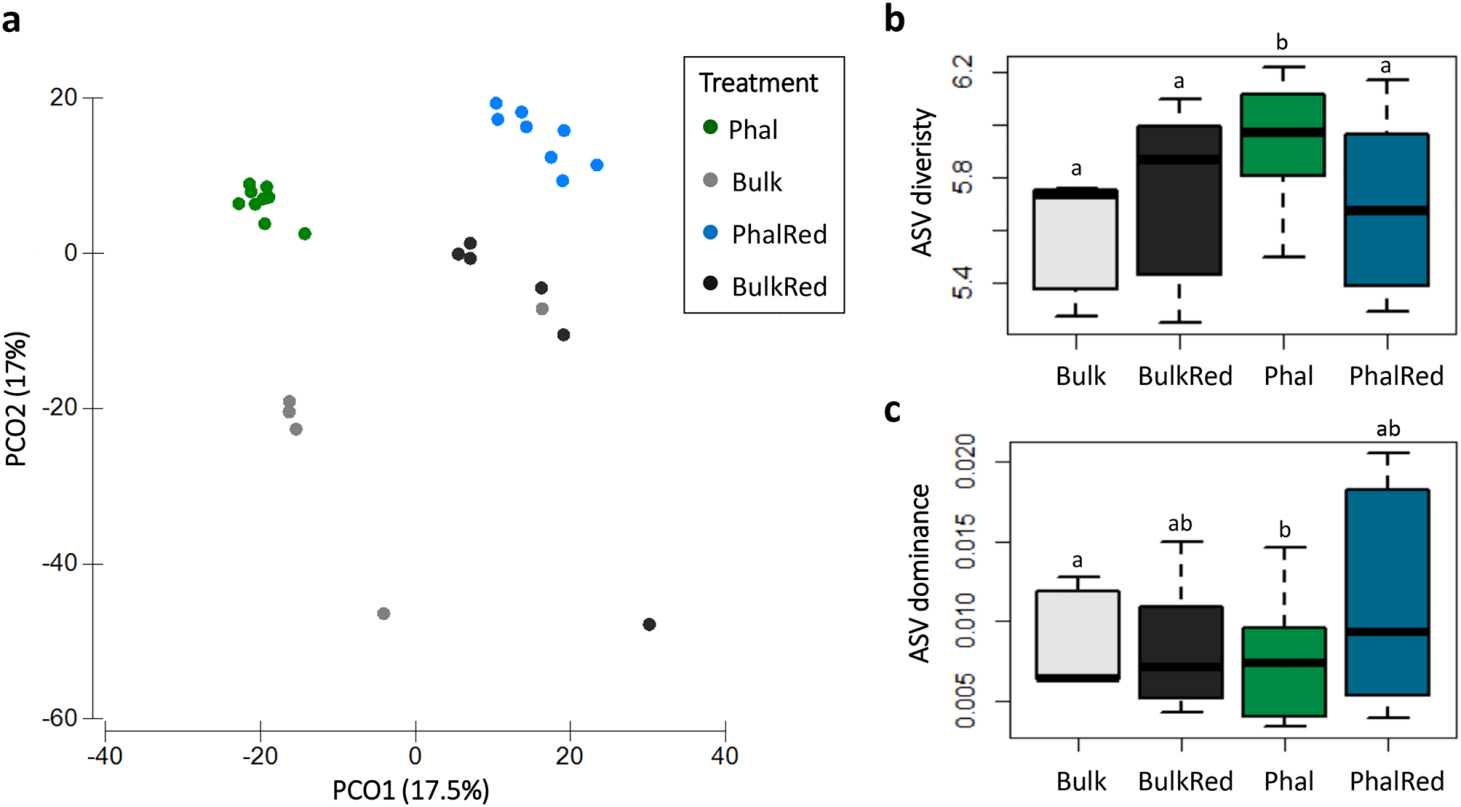
Analysis of bacterial community’s structure and diversity of soil samples after DNA fractioning and sequencing of the main 16S rRNA peak from T_0_ and unlabeled SIP incubations. (**a**) Principal coordinates analysis of the ASVs obtained from the bacterial communities according to the different soil treatments. (**b**) ASVs alpha diversity according to Shannon and (**c**) Dominance diversity indices. Letters indicate the statistical differences among time points, according to the analysis of variance (ANOVA).

Following PERMDISP validation (*p *= 0.179), the beta-diversity analysis on the ASV dataset showed that bacterial communities of soils subjected to different treatments grouped separately as represented by the PCoA (Fig. 2A) and differed significantly from one another as demonstrated by CAP (delta_1^2: 0.98974, *p *= 0.0001) and PERMANOVA main and pairwise tests, with the exception of the two non-planted soils (Supplementary Table S4A-B). Beta-diversity based on ASV composition according to the time of SIP incubation cannot be discussed due to the difference in dispersion among soil samples of T_0_, D_21,_ and D_28_ (PERMDISP: *p *= 0.021), however we could observe a diversity given by this factor when considered together with the soil treatment (PERMDIS* p *= 0.142; PERMANOVA: F_2,6_ = 2.46, *p *= 0.0001). Estimation of the factors’ contributions to the observed variations determined by PERMANOVA showed that “treatment” alone explained 28% of the variation and an additional 23% was explained by its interaction with the factor “time” (Supplementary Table S4C). Furthermore, PCoA and PERMANOVA (Supplementary Figure S1; Supplementary Table S5) separated the communities of planted soils from the bulk soils (PERMDISP: *p *= 0.072), and those of soils subjected to a redox cycle from those that were not (PERMDISP: *p *= 0.748).

Bacterial alpha-diversity was affected by different soil treatments, with Phal soils hosting the most diverse community compared to the other treatments, as shown by the Shannon index and demonstrated by ANOVA (Fig. 2B). Soils planted with *P. arundinacea* and not subjected to redox cycle (Phal) were also characterized by lower dominance, even though the difference was statistically significant only compared to the non-planted Bulk control (Fig. 2C). The analysis of the alpha-diversity also showed a decrease in the Shannon index over time, while the dominance index followed an opposite trend. However, both the parameters differed significantly only between pre-incubation (T_0_) and SIP-incubated soils (D_21_, D_28_) according to two-way ANOVA (Supplementary Table S6).

The main bacteria phyla, having relative abundance between 25 and 5%, observed in the total community of pre-incubation samples (T_0_) were Proteobacteria, Actinobacteria, Acidobacteria, Chloroflexi and Planctomycetes. The different soil treatments presented a different profile in terms of phyla/class relative abundance (Supplementary Figure S2). ASVs affiliated with Actinobacteria were dominant in soil planted with *P. arundinacea* (Phal) (25% on average between the replicates), while they declined in non-planted soil samples (Bulk, 15.5%) and in both planted and non-planted soils subjected to redox cycle (PhalRed, 13.2% and BulkRed, 9.2%). ASVs affiliated with Proteobacteria showed similar abundance in all the T_0_ soil samples but were mainly represented by Alphaproteobacteria in Phal (11.9%) and Bulk (16.7%) soils, and by Betaproteobacteria in the repeatedly flooded soils (Phalred and BulkRed, 10% on average). Chloroflexi were enriched in PhalRed and BulkRed soils, representing respectively 20.5% and 19.8% of the total community. Other less abundant taxa whose relative abundance changed with soil treatments were Firmicutes, which were reduced in plant-biostimulated soils, and Desulfobacterota, which were increased in soils subjected to redox conditions.

After incubation with 4-chlorobiphenyl, ASVs affiliated with Proteobacteria became prevalent, representing the 30% of the communities of Phal and PhalRed soils at D_21_ and further increasing up to 40% in Phal soils at D_28_. Actinobacteria were more abundant in PhalRed soils at D_28_ compared to T_0_ while the abundance of Chloroflexi was decreased in all the D_28_ communities.

### Identification of bacterial taxa deriving carbon from 4-chlorobiphenyl

**Figure 3:**
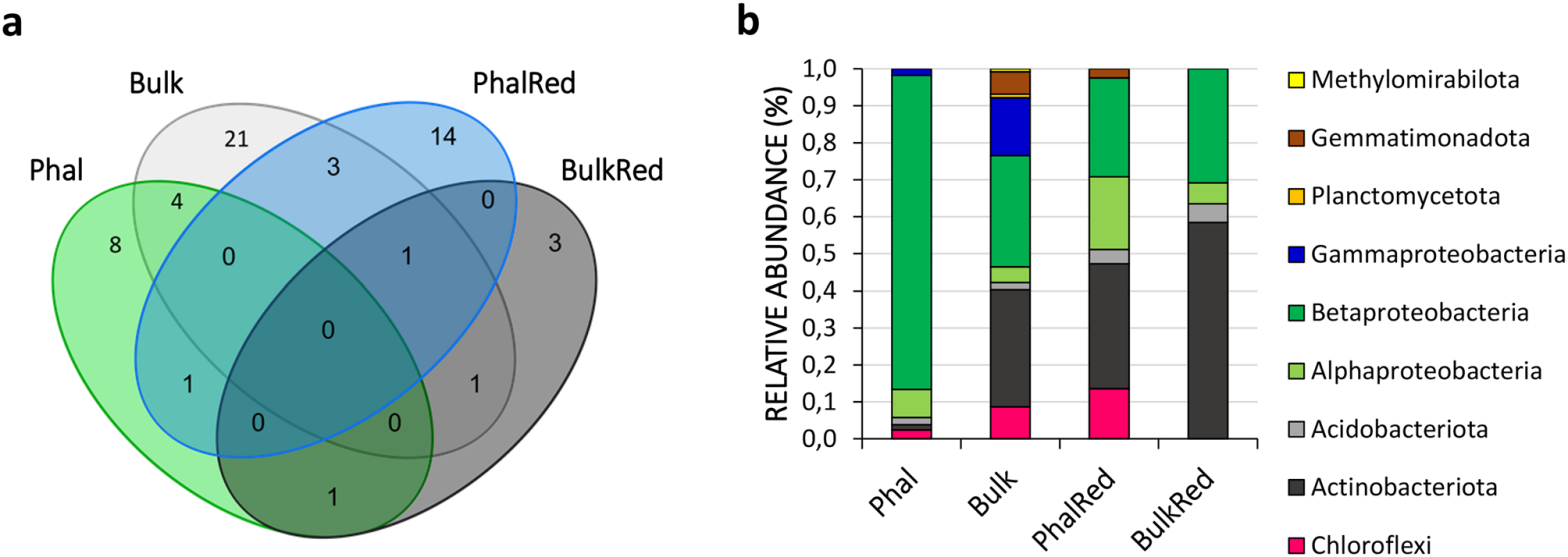
Distribution of ASVs and taxonomic diversity of bacterial populations incorporating ^13^C from labeled 4-chlorobiphenyl associated to the different soils after twenty-eight days of SIP incubation. (**a**) Venn diagrams showing the shared and exclusive bacterial ASVs of the soils from the four different rhizoremediation treatments (Phal, Bulk, PhalRed, BulkRed). (**b**) Relative abundance of different bacterial phylum/class incorporating ^13^C in the labeled D_28_ microcosms.

Fifty-seven different ASVs corresponding to bacterial taxa incorporating ^13^C derived from the mineralization of 4-CB were identified in D_28_ soils after rarefaction (Supplementary table S7A). We observed the highest number of ASVs (n = 30) in Bulk soils, followed by PhalRed (n = 19), Phal (n = 14), and BulkRed (n = 6). Most of these ASVs (n = 46) were unique to the different soil treatments and none was shared among all of them (Fig. 3A). Only one ASV, affiliated with the genus *Rhodococcus*, was common in three of the considered soils (i.e., Bulk, PhalRed and BulkRed).

Within the bacteria deriving carbon from 4-CB, the most abundant class/phyla in all four treated soils were Betaproteobacteria and Actinobacteria (Fig. 3B). In particular, the bacterial ASVs detected in Phal soils mostly belonged to the family Comamonadaceae (85.7% of the ^13^C-incorporating bacteria), which represented only 20% in Bulk and 24.3% in PhalRed soils and were absent in BulkRed soils (Table 1). Instead, we found Rhodocyclaceae among the ^13^C-incorporating bacteria only in non-planted soils (4.2% in Bulk and 30.8% in BulkRed). Among Actinobacteria, Nocardiaceae were represented only by ASV3, and were particularly abundant in Bulk and BulkRed soils (28.1% and 58.4% respectively), while in planted soils they represented only 2.6% in PhalRed and were absent in Phal. PhalRed soil bacteria that derived ^13^C from 4-CB were affiliated with other Actinobacteria families, such as Pseudonocardiaceae (18.3%), Micromonosporaceae (8.1%), and Nocardioidaceae (4.7%). Alphaproteobacteria deriving carbon from 4-CB were also retrieved in all the soils but were more abundant in planted (7.7% in Phal and 19.7% in PhalRed) than in non-planted soils (4.3% in Bulk and 5.7% in BulkRed). The most represented families within this clade included mainly Dongiaceae (7.8%) and other unidentified taxa (6.7%) in PhalRed soils and Sphingomonadaceae in BulkRed (5.7%). Other phyla that were identified to have derived ^13^C from 4-CB were Chloroflexi (13.6% in PhalRed, 8.7% in Bulk and 2.4% in Phal samples) and Acidobacteria (5.0% in BulkRed, 3.9% in PhalRed and 1.9% in both Phal and Bulk samples). Considering the relative abundance of ASVs corresponding to ^13^C-deriving bacteria in the total community of each sample (i.e., 4,000 reads obtained after rarefaction) and comparing it with the relative abundance of the same ASVs in T_0_ soil bacterial communities, we could infer that the most relevant families identified herein as deriving carbon from 4-CB were enriched during the SIP incubation period (Supplementary Table S8).

**Table 1:** Bacteria families incorporating ^13^C derived from the mineralization of labeled 4-chlorobiphenyl identified after twenty-eight days (D_28_) of SIP incubation.

Phylum	Class	Order	Family	Number of ASV (Mean)				Relative abundance (%)			
				Phal	Bulk	PhalRed	BulkRed	Phal	Bulk	PhalRed	BulkRed
Acidobacteria	Holophagae	NA Holophagae	NA Holophagae	–	25	–	–		1.9		
Acidobacteria	Vicinamibacteria	NA Vicinamibacteria	NA Vicinamibacteria	–	–	–	11	–	–	–	2.1
Acidobacteria	Vicinamibacteria	Vicinamibacterales	NA Vicinamibacterales	10	0	40	16	0.9	–	3.0	2.9
Acidobacteria	Vicinamibacteria	Vicinamibacterales	Vicinamibacteraceae	11	–	11	–	1.0	–	0.9	–
Actinobacteria	Actinobacteria	Corynebacteriales	Nocardiaceae	0	376	34	313	–	28.1	2.6	58.4
Actinobacteria	Actinobacteria	Frankiales	Sporichthyaceae	16	0	–	–	1.4	–	–	–
Actinobacteria	Actinobacteria	Micromonosporales	Micromonosporaceae	–	–	107	–	–	–	8.1	–
Actinobacteria	Actinobacteria	Propionibacteriales	Nocardioidaceae	–	16	62	–	–	1.2	4.7	–
Actinobacteria	Actinobacteria	Pseudonocardiales	Pseudonocardiaceae	–	–	242	–	–	–	18.3	–
Actinobacteria	Thermoleophilia	Gaiellales	Gaiellaceae	–	32	–	–	–	2.4	–	–
Chloroflexi	Anaerolineae	Anaerolineales	Anaerolineaceae	11	83	–	–	–	6.2	–	–
Chloroflexi	Anaerolineae	Caldilineales	Caldilineaceae	–	–	38	–	–	–	2.9	–
Chloroflexi	Anaerolineae	NA Anaerolineae	NA Anaerolineae	16	–	–	–	1.4	–	–	–
Chloroflexi	Chloroflexia	Thermomicrobiales	NA Thermomicrobiales	–	17	21	–	–	1.3	1.6	–
Chloroflexi	NA Chloroflexi	NA Chloroflexi	NA Chloroflexi	0	16	121	0		1.2	9.1	
Gemmatimonadetes	Gemmatimonadetes	Gemmatimonadales	Gemmatimonadaceae	–	–	33	–	–	–	2.5	–
Gemmatimonadetes	Longimicrobia	Longimicrobiales	Longimicrobiaceae	–	66	–	–	–	4.9	–	–
Gemmatimonadetes	NA Gemmatimon	NA Gemmatimon	NA Gemmatimon	–	15	–	–	–	1.1	–	–
Methylomirabilota	Methylomirabilia	Rokubacteriales	Rokubacteriales NA	–	11	–	–	–	0.8	–	–
Planctomycetes	NA Planctomycetes	NA Planctomycetes	NA Planctomycetes	–	13	–	–	–	1.0	–	–
Proteobacteria	Alphaproteobacteria	Acetobacterales	Acetobacteraceae	–	27	–	–	–	2.0	–	–
Proteobacteria	Alphaproteobacteria	Azospirillales	Azospirillaceae	–	18	–	–	–	1.3	–	–
Proteobacteria	Alphaproteobacteria	Dongiales	Dongiaceae	–	–	104	–	–	–	7.8	–
Proteobacteria	Alphaproteobacteria	NA Alphaproteob	NA Alphaproteob	–	–	89	–	–	–	6.7	–
Proteobacteria	Alphaproteobacteria	Rhizobiales	Devosiaceae	21	–	–	–	1.9	–	–	–
Proteobacteria	Alphaproteobacteria	Rhizobiales	Methyloligellaceae	18	–	–	–	1.7	–	–	–
Proteobacteria	Alphaproteobacteria	Rhizobiales	Rhizobiaceae	–	–	44	–	–	–	3.3	–
Proteobacteria	Alphaproteobacteria	Rhizobiales	Xanthobacteraceae	30	–	–	–	2.7	–	–	–
Proteobacteria	Alphaproteobacteria	Rhodobacterales	Rhodobacteraceae	–	13	–	–	–	1.0	–	–
Proteobacteria	Alphaproteobacteria	Sphingomonadales	Sphingomonadaceae	15	–	24	31	1.4	–	1.8	5.7
Proteobacteria	Betaproteobacteria	Burkholderiales	Comamonadaceae	925	267	321	–	85.7	20.0	24.3	–
Proteobacteria	Betaproteobacteria	Burkholderiales	Nitrosomonadaceae	–	80	32	–	–	5.9	2.4	–
Proteobacteria	Betaproteobacteria	Burkholderiales	Rhodocyclaceae	–	37	–	165	–	4.2	–	30.8
Proteobacteria	Gammaproteobacteria	Pseudomonadales	Pseudomonadaceae	–	189	–	–	–	14.1	–	–
Proteobacteria	Gammaproteobacteria	Salinisphaerales	Solimonadaceae	19	–	–	–	1.8	–	–	–
Proteobacteria	Gammaproteobacteria	Steroidobacterales	Steroidobacteraceae	–	21	–	–	–	1.5	–	–

The table reports the mean number of ASVs retrieved for each bacteria family in the biological replicates of the four original soil treatments (Phal, Bulk, PhalRed and BulkRed) and the relative abundance over the ^13^C-incorporating population.

## Discussion

Rhizoremediation through plant biostimulation of autochthonous microbial degraders is considered an environmentally sustainable and cost-effective promising technology for the clean-up of weathered PCB-polluted soils^8^. However, plant species-specific rhizosphere effect together with soil treatment practices, differentially impact soil bacterial communities, which further affects pollutants’ depletion rates^6,27,28^. Consequently, a lack of knowledge still exists about how biodegradation of PCB congeners occurs and which are the main microbial players under different soil rhizoremediation treatments. In this work, a historically contaminated soil containing an initial microbiome naturally selected during twelve years of natural attenuation^17^ was biostimulated for 18 months using reed canary grass and/or redox cycle, aiming at the enrichment of microbial degraders. Subsequent soil incubation with ^13^C-4-CB demonstrated the presence of bacteria that were able to assimilate 4-CB.

The results of the evolution of ^13^CO_2_ in soil microcosms showed that after one week of SIP incubation there was no mineralization of the labeled substrate regardless of the original soil treatment, and, apart from two D_21_ samples, we were able to detect ^13^C labeled DNA only after twenty-eight days (D_28_). These results are in contrast with the only previous studies that used 4-CB as a metabolic tracer of PCB biodegradation in a stream sediment^16,29^, in which the substrate mineralization and the identification of bacterial degraders occurred after one to seven days of incubation. Possible explanations could be that the different used matrices are characterized by diverse native bacterial communities together with environmental conditions and pollution profiles that can differentially impact the biodegradation rate, as shown for biphenyl by Chen and co-authors^30^. These differences may reflect in the incorporation of ^13^C by bacterial cells and consequently in DNA-labelling: for instance, in other works labeled DNA was detected only after fourteen days of incubation with biphenyl^31,32^. As 4-CB has a slower depletion rate compared to biphenyl due to the presence of chlorine^33^, it is possible that the conditions of the soil samples used in this study, such as the presence of other classes of contaminants in addition to PCBs^34^, determined a longer time for 4-CB mineralization and for the incorporation of derived carbon into bacterial DNA.

The results of 16S rRNA amplicon sequencing from the DNA of the T_0_ soils before the 4-CB microcosm enrichment, confirmed that the applied treatments, i.e. biostimulation, periodic flooding, and their combination, induced the differentiation of the structure and taxonomic composition of the total bacterial community, corroborating previous information on beta-diversity obtained by DNA fingerprinting targeting the 16S rRNA- 23S rRNA intergenic spacers^12^. Considering the dominant taxa, plant biostimulation with *P. arundinacea* induced an increase of Actinobacteria, though this effect was partially attenuated by the soil flooding. The phyla Chloroflexi and Desulfobacterota (formerly included in the class Deltaproteobacteria^35^) were significantly enriched in soils subjected to the redox cycle, suggesting the occurrence of reductive dechlorination metabolisms, potentially able to reduce the concentration of highly chlorinated molecules. Members of dehalorespiring taxa, such as *Dehalococcoides* and *Geobacter*, have been in fact studied for their PCB dehalogenation activity^36,37^. Besides the effect exerted by soil treatments on the original soil microbiota, our data also report a shift towards less diverse bacterial communities over SIP incubation time in terms of alpha-diversity, with the enrichment of Proteobacteria and Actinobacteria, putatively driven by the incubation condition.

The combination of SIP and 16S rRNA gene sequencing allowed us to identify the ASVs deriving carbon from 4-CB at the end of SIP incubation (D_28_). The bacterial cells incorporating ^13^C may have acquired carbon both through direct degradation of 4-CB and via cross-feeding on catabolic intermediates or other metabolites. In a time-flow experiment with parallel incubation using biphenyl or benzoate as substrates^32^ it was suggested that different taxa are responsible for either the upper or lower PCB degradation pathways, even though a partial overlap in the two communities may include also bacteria performing the whole pathway. The dataset generated in this study does not allow for a clear discrimination between the two groups, however the results are consistent with previous DNA-SIP studies, where the predominant taxa detected also herein, such as Comamonadaceae, Rhodocyclaceae, Nocardiaceae and Pseudonocardiaceae, were identified as biphenyl and/or 4-CB degraders in PCB contaminated soils and sediments^16,18,29,38,39^. The prevalent ASVs belonged to Comamonadaceae and were classified as *Hydrogenophaga* or *Caenimonas* in Phal and Bulk soil samples, and as *Methylibium* in PhalRed ones. In addition to the above-mentioned DNA-SIP works, *Hydrogenophaga* sp. has also been characterized as a PCB degrader by Lambo and Patel^40^ whereas *Caenimonas* and *Methylibium* were associated with the degradation of aromatic compounds in soil and groundwater^41–43^ and may therefore be cross feeders on PCB catabolic intermediates. Among Rhodocyclaceae, *Azoarcus* sp., a genus that we found abundant among ^13^C-labeled ASVs in Bulk soils, was also identified as capable of oxidizing biphenyl in upland soil by a recent study based on a protein-SIP approach^30^. Considering the family Nocardiaceae, *Rhodococcus* strains are well known PCB degraders and have been shown to carry out different degradation pathways from biphenyl to benzoate in a metagenomic study conducted on an enrichment culture growing on biphenyl^44^. Interestingly, in the present study, the only ASV representing this bacterial family was common to three of the considered soils (Bulk, BulkRed and PhalRed) and was classified as *Rhodococcus*. Three *Rhodococcus* sp. strains were indeed previously isolated from this soil after three months of rhizoremediation treatment with reed canary grass and a redox cycle (PhalRed), and their capacity to utilize biphenyl as a sole carbon source and to degrade different PCB congeners present in the Delor 103 commercial mixture was demonstrated in vitro through a resting-cell assay^45^. Therefore, we can hypothesize that this genus was involved in the direct degradation of 4-CB in the present study as well as in the degradation of other PCB congeners in this historically polluted soil.

Although most of the ASVs derived from D_28_ 4-CB amended microcosms were unique to each of the four soil treatments, we could not observe the same pattern comparing their relative abundance in initial T_0_ soils. In fact, in pre-incubation samples, the same ASVs were mostly below or close to the detection threshold, indicating that the enrichment of the detected degraders occurred over time during the incubation in microcosms supplemented with 4-CB This may imply that, likewise the total bacterial community, the alpha-diversity of active PCB-degraders was higher in the initial soils subjected to the rhizoremediation treatments, with active taxa residing in the rare biosphere. Only ASV6 within Comamonadaceae, the prevalent taxon incorporating carbon from 4-CB in Phal soil at D_28,_ even though representing less than 1% of the bacterial communities before SIP incubation, seemed to be enriched in previously planted treatments compared to bulk soils at T_0_. This taxon was already observed to be positively selected by plant species within the Poaceae family, both in uncontaminated and polluted soils^46,47^ and to be associated with an increase in organic pollutant biodegradation^48^. It is possible that the predominance of ASV6 among degraders in Phal soils was favored by an efficient capacity to utilize the compounds derived from root deposition that may be implied in the co-metabolism of 4-CB or its catabolic intermediates^5^. Instead, among Comamonadaceae in PhalRed soils we observed the enrichment of ASV4 classified as *Methylibium*, a facultative aerobe previously described for PAH anaerobic degradation^43^, that could have been favored in this partially anoxic environment.

In a former study, we showed that rhizoremediation with *P. arundinacea* alone or in combination with soil flooding resulted in a significant reduction in the concentration of low chlorinated PCBs (≤ pentaCBs) by stimulating microbial activity and inducing a shift in the overall soil bacterial community structure^12^. In this study, we described at taxonomic level how these treatments shaped the structure of soil bacterial communities and we observed that Actinobacteria were enriched by the plant biostimulation. Using 4-CB as a metabolic tracer for DNA Stable Isotope Probing (SIP), we also provided new insights into the populations metabolically active in PCB degradation, identifying members of Actinobacteria and Betaproteobacteria as the main players involved. Altogether this information pointed out that Actinobacteria and Betaproteobacteria are the key bacterial targets to be enriched in a plant biostimulation-based approach aimed at the on-site bioremediation of the highly and historically PCB-polluted site considered in our study. Future research perspectives include in situ-SIP coupled with metagenomic analysis^49^ and will allow to deepen the investigation of plant-microbiome interactions and increase the knowledge of the metabolic reactions occurring during the PCB biodegradation in the field.

## Supplementary Information

Supplementary Information 1.

Supplementary Information 2.

Supplementary Information 3.

Supplementary Information 4.

## Data Availability

All relevant data are within the paper and its Supporting Information files. The 16S rRNA gene sequence reads were deposited in the NCBI SRA database under the BioProject ID: PRJNA809320 as indicated in the Materials and Methods section.
