# Supplementary material for: DNA stable isotope probing on soil treated by plant biostimulation and flooding revealed the bacterial communities involved in PCB degradation
Source: Sci Rep. 2022 Nov 10;12:19232. doi: 10.1038/s41598-022-23728-2 (PMC9649793; doi:10.1038/s41598-022-23728-2)

SUPPLEMENTARY INFORMATION

DNA stable isotope probing on soil treated by plant biostimulation and flooding revealed the bacterial communities involved in PCB degradation

Lorenzo Vergani[1], Francesca Mapelli[1], Magdalena Folkmanova[2], Jakub Papik[2], Jan Jansa[3] Ondrej Uhlik[2], Sara Borin[1]*

[1]Department of Food, Environmental and Nutritional Sciences, University of Milan, Milan, Italy
[2]Departement of Biochemistry and Microbiology, University of Chemistry and Technology Prague, Prague, Czech Republic
[3]Laboratory of Fungal Biology, Institute of Microbiology, Czech Academy of Sciences, Prague, Czech Republic

*sara.borin@unimi.it

Supplementary Table S1. Amounts of $^{13}CO_2$ evolved for each biological replicate after 7, 21 and 28 days of incubation of the soil samples with ^{13}C-4-chlorobiphenyl and percentage amounts of original substrate which has been converted to $^{13}CO_2$.

Treatment	Replicate	Time	Total CO_2 (µM)	$^{13}CO_2$ µM	Mineralized 4-CB (%)
Phal	1	D07	2.42E+02	8.62E-01	2.71
Phal	2	D07	2.00E+02	4.04E-01	1.27
Phal	3	D07	1.87E+02	2.78E-01	0.87
Bulk	1	D07	8.44E+01	-7.88E-01	-2.48
Bulk	2	D07	1.46E+02	-1.57E-01	-0.49
Bulk	3	D07	1.58E+02	-2.84E-02	-0.09
PhalRed	1	D07	1.19E+02	-3.21E-01	-1.01
PhalRed	2	D07	1.13E+02	-4.68E-01	-1.47
PhalRed	3	D07	1.00E+02	-5.58E-01	-1.76
BulkRed	1	D07	6.73E+01	2.64E-01	0.83
BulkRed	2	D07	4.79E+01	-1.05E+00	-3.29
BulkRed	3	D07	1.08E+02	-4.15E-01	-1.30
Phal	1	D21	1.83E+02	6.54E+00	20.56
Phal	2	D21	2.11E+02	3.93E+00	12.35
Phal	3	D21	1.95E+02	1.12E+00	3.53
Bulk	1	D21	1.51E+02	1.45E+00	4.56
Bulk	2	D21	1.01E+02	1.76E+00	5.54
Bulk	3	D21	1.39E+02	6.12E+00	19.24
PhalRed	1	D21	1.27E+02	3.82E+00	12.01
PhalRed	2	D21	1.06E+02	7.92E+00	24.89
PhalRed	3	D21	1.17E+02	8.13E+00	25.57
BulkRed	1	D21	1.13E+02	6.81E+00	21.40
BulkRed	2	D21	1.04E+02	6.65E+00	20.90
BulkRed	3	D21	1.03E+02	7.08E+00	22.25
Phal	2	D28	1.91E+02	1.45E+01	45.63
Phal	3	D28	2.12E+02	3.75E+00	11.80
Bulk	1	D28	1.71E+02	7.18E+00	22.57
Bulk	2	D28	1.18E+02	7.22E+00	22.69
Bulk	3	D28	1.12E+02	9.76E+00	30.69
PhalRed	1	D28	1.50E+02	1.03E+01	32.34
PhalRed	3	D28	1.42E+02	1.48E+01	46.52
BulkRed	1	D28	1.67E+02	9.98E+00	31.38
BulkRed	2	D28	1.52E+02	8.01E+00	25.19
BulkRed	3	D28	1.47E+02	1.41E+01	44.47

Supplementary Table S2 A-F. Provided as a separate excel file. Buoyant density (BD) and 16S rRNA gene copies number (SQ) reported for each fraction belonging to the density gradients of each replicate sample. Plots represent normalized SQ as a function of BD. In each gradient, fractions that were determined to contain ^{13}C-labeled DNA based on quantities of DNA and subsequently combined into pools are highlighted. Equivalent pools were prepared for controls incubated with unlabeled substrates. The fraction composition, SQ and BD mean values of each pool are summarized in a table for each gradient.

Supplementary Table S3. Provided as a separate excel file. A) List of the DNA samples sequenced and their correspondence with different SIP incubation substrate, gradient's peak, incubation time and original soil treatments. **B)** List of ASVs present in the DNA samples, and number of corresponding reads, after sequence rarefaction.

Supplementary Table S4. PERMANOVA for the 16S rRNA gene-based Illumina dataset. A) Main test comparison of the distance matrix generated according to ASVs distribution in soils subjected to the four treatments (Phal, Bulk, PhalRed, BulkRed), different SIP incubation times (T_0, D_{21}, D_{28}) and their interaction using PERMANOVA. **B)** Pair-wise test comparison between the different soil treatments. **C)** Estimates of component variation considering the factors "time" and "treatment". In bold the statistically significant terms (Supplementary Table4A).

**A) PERMANOVA table of results**							
Source	df	SS	MS	Pseudo-F	P(perm)	perms	P(MC)
time	2	8269	4134.5	5.1572	0.0001	9893	0.0001
treatment	3	17172	5723.9	7.1397	0.0001	9903	0.0001
timextreatment	6	11824	1970.6	2.458	0.0001	9789	0.0001
Res	16	12827	801.7				
Total	27	48332					
**B) Pair-wise test (treatment)**							
Groups	t	P(perm)	perms	P(MC)			
Phal, PhalRed	2.7427	0.0001	8157	0.0001			
Phal, Bulk	2.266	0.0006	1980	0.0009			
Phal, BulkRed	2.289	0.0005	4319	0.001			
PhalRed, Bulk	2.3047	0.0004	1282	0.0006			
PhalRed, BulkRed	1.6924	0.0004	2895	0.0125			
Bulk, BulkRed	1.304	0.0563	462	0.1451			
**C) Estimates of components of variation**							
Source	Estimate	Sq.root					
S(time)	421.17	20.522					
**S(treatment)**	**800.95**	**28.301**					
**S(timextreatment)**	**546.38**	**23.375**					
V(Res)	801.7	28.314					

Supplementary Table S5. PERMANOVA Pair-wise test comparison between different original soil conditions. A) Comparison between planted and non-planted soils. **B)** Comparison between soils subjected or not to the redox cycle.

**A) Term 'biostimulation'**				
Groups	t	P(perm)	perms	P(MC)
planted, no plant	2,1197	0,0001	9882	0,0001
**B) Term 'redox'**				
Groups	t	P(perm)	perms	P(MC)
no redox, redox	2,1512	0,0001	9898	0,0001

Supplementary Table S6. Analysis of Variance (ANOVA) of Shannon diversity and Dominance indices calculated from the ASVs table. A) Main test comparison considering the factors "time", "treatment" and their interaction using two-way ANOVA. **B)** Pair-wise test comparison between the different time points. **C)** Pair-wise test comparison between the different soil treatments.

**A) Analysis of Variance Table: p-value**		
Response:	Shannon	Dominance
time	0.0005	0.0000
treat	0.0025	0.0228
time:treat	0.3806	0.6563
**B) Tukey pair-wise test (time): p-value**		
Response:	Shannon	Dominance
D21-T0	0.0154	0.0000
D28-T0	0.0006	0.0000
D28-D21	0.1777	0.2689
**C) Tukey pair-wise test (treatment): p-value**		
Response:	Shannon	Dominance
BulkRed-Bulk	0.7860	0.9880
Phal-Bulk	0.0036	0.0499
PhalRed-Bulk	0.3800	0.9346
Phal-BulkRed	0.0309	0.0987
PhalRed-BulkRed	0.9268	0.9955
PhalRed-Phal	0.0509	0.0823

Supplementary Table S7. Provided as a separate excel file. ASVs table corresponding to bacteria taxa incorporating ^{13}C derived from the mineralization of 4-chlorobiphenyl. A) ASVs detected at D_{28}. For each soil treatment (Phal, Bulk, PhalRed and BulkRed) the mean number of each ASV retrieved in the biological replicates is reported together with its relative abundance over the total degraders. **B)** ASVs detected in D_{21} samples Bulk 3 and BulkRed 2

Supplementary Table S8. Relative abundance of ASVs deriving carbon from 4-CB over the total bacteria community. The table shows the percentage of most abundant ^{13}C-incorporating ASVs identified after SIP incubation at D_{28} over the total number of reads obtained after rarefaction (n=4000), compared with their percentage in the original soil samples at initial $T_{0.}$

			**T0**				**13C-Incorporating D28**			
**Family**	**Genus**	**ASV**	**Phal**	**Bulk**	**PhalRed**	**BulkRed**	**Phal**	**Bulk**	**PhalRed**	**BulkRed**
Comamonadaceae	Hydrogenophaga	ASV12	-	-	-	-	4,80	5,48	-	-
Comamonadaceae	Hydrogenophaga	ASV2	-	-	-	-	6,45	-	-	-
Comamonadaceae	Methylibium	ASV4	0,03	-	-	-	-	-	8,03	-
Comamonadaceae	Caenimonas	ASV6	0,38	0,02	0,63	0,16	9,29	-	-	-
Rhodocyclaceae	Azoarcus	ASV11	0,06	0,00	0,40	0,09	-	1,39	-	2,79
Rhodocyclaceae	Azoarcus	ASV43	-	-	-	-	-	-	-	1,33
Pseudomonadaceae	Pseudomonas	ASV104	-	-	-	-		4,71		
Nocardiaceae	Rhodococcus	ASV3	-	-	0,03	-	-	9,39	0,85	7,83
Pseudonocardiaceae	Pseudonocardia	ASV1	-	-	-	-			6,05	

Supplementary Figure S1. Principal coordinates analysis of the ASVs obtained from the bacterial communities according to the different soil treatments divided as **A)** presence of plant biostimulation and **B)** induction of a redox cycle.

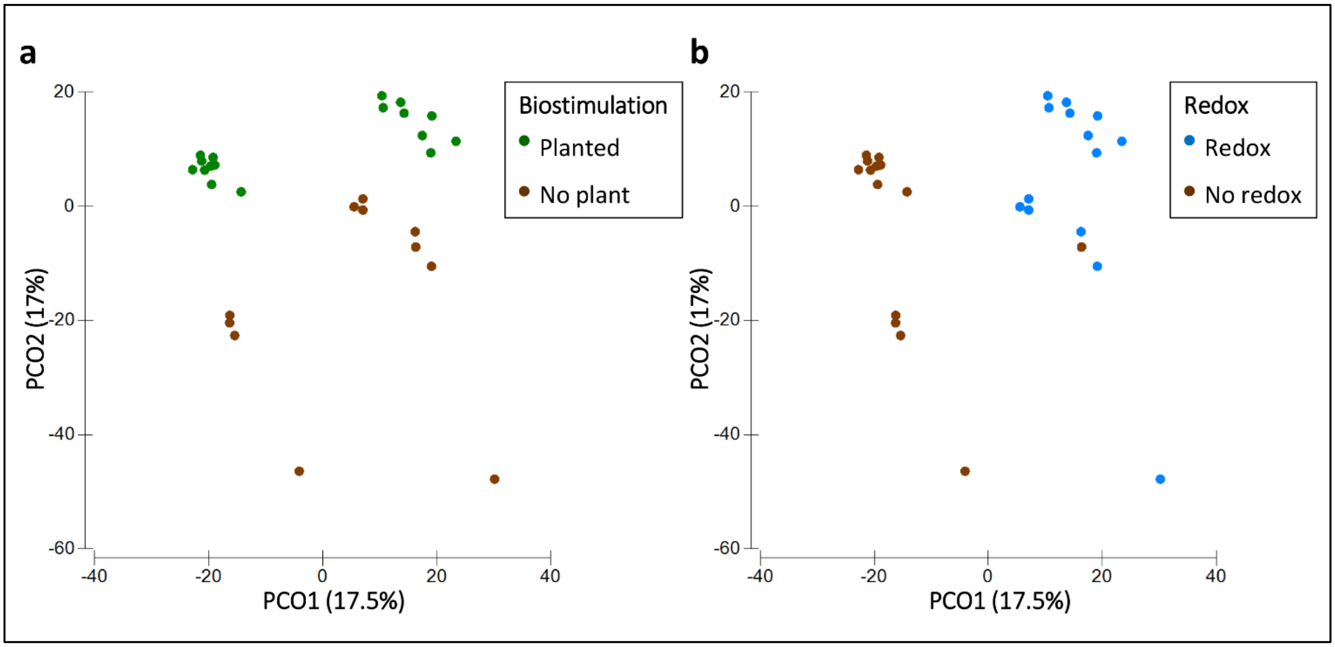

Supplementary Figure S2. Relative abundance of different bacterial phylum/class in the soil samples after DNA fractionation and sequencing of the main peak from T_0 and unlabeled SIP incubations at the time points D_{21} and D_{28}. Phyla/classes representing less than 1% out of total reads were grouped as 'Others.'

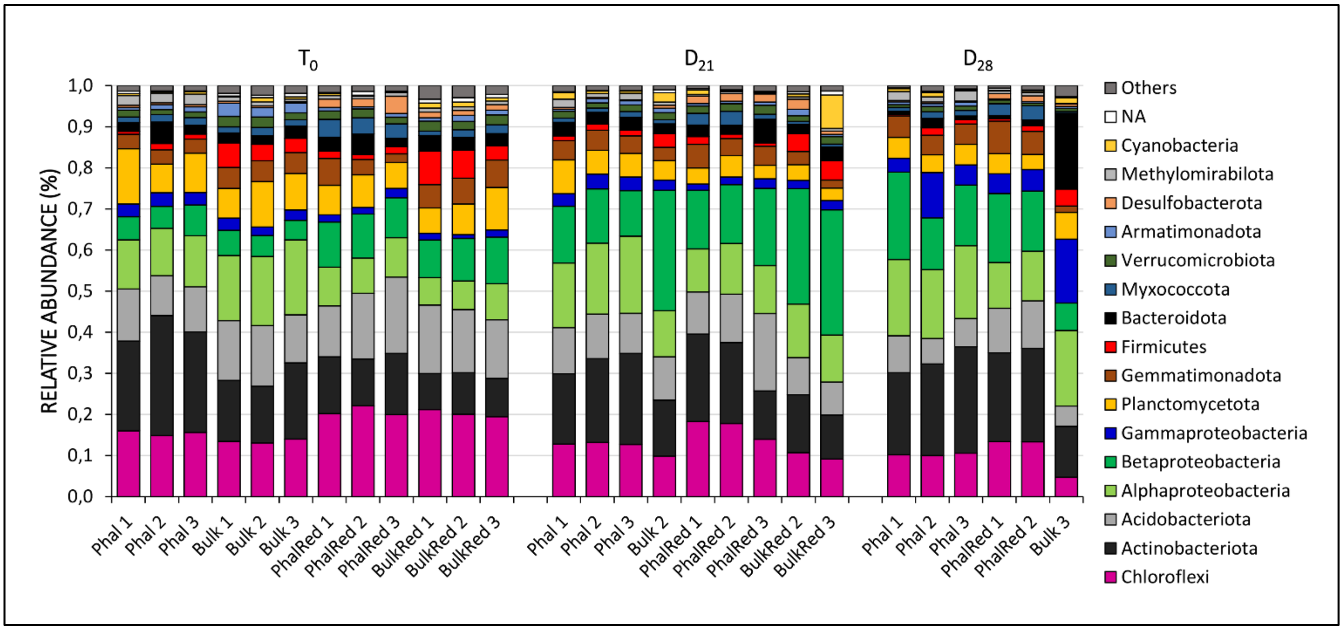

Supplement: Supplementary file 1 — Supplementary Information 1. [file 41598_2022_23728_MOESM1_ESM.pdf]
